# Alpha-Melanocyte Stimulating Hormone: An Emerging Anti-Inflammatory Antimicrobial Peptide

**DOI:** 10.1155/2014/874610

**Published:** 2014-07-23

**Authors:** Madhuri Singh, Kasturi Mukhopadhyay

**Affiliations:** ^1^School of Environmental Sciences, Jawaharlal Nehru University, New Delhi 110067, India; ^2^School of Life Sciences, Jawaharlal Nehru University, New Delhi 110067, India

## Abstract

The alpha-melanocyte stimulating hormone (*α*-MSH) is a neuropeptide belonging to the melanocortin family. It is well known for its anti-inflammatory and antipyretic effects and shares several characteristics with antimicrobial peptides (AMPs). There have been some recent reports about the direct antimicrobial activity of *α*-MSH against various microbes belonging to both fungal and bacterial pathogens. Similar to *α*-MSH's anti-inflammatory properties, its C-terminal residues also exhibit antimicrobial activity parallel to that of the entire peptide. This review is focused on the current findings regarding the direct antimicrobial potential and immunomodulatory mechanism of *α*-MSH and its C-terminal fragments, with particular emphasis on the prospects of *α*-MSH based peptides as a strong anti-infective agent.

## 1. Introduction

*α*-MSH is an endogenous neuropeptide derived from proopiomelanocortin (POMC), a common precursor protein of all melanocortin peptides, which expresses in the pituitary gland [1, 2]. It is primarily a pigmentary hormone of the vertebrates and largely influences immune reactions in the host for controlling inflammation in the brain and peripheral organs [3, 4]. Both* in vitro* and* in vivo* studies have confirmed that, inside the host, *α*-MSH reduces the concentration of proinflammatory mediators through the induction of cyclic adenosine monophosphate (cAMP) and inhibition of the nuclear factor *κβ* (NF-*κβ*), thus protecting the brain and peripheral organs from inflammatory disorders [5–7].

The presence of the ancestral components of host immunity—AMPs—has been reported across all classes including human, and their immunomodulatory role has been widely implicated in fighting against infection [8–10]. Similar to AMPs, *α*-MSH is a short peptide, cationic in nature. Although the primary site of *α*-MSH expression is pituitary gland, its presence in defense cells and several peripheral sites including skin pointed towards its immunoregulatory properties [11]. These characteristics of *α*-MSH openly suggest its role in host immunity, specifically innate immunity, and provide a platform to look for its direct antimicrobial properties [12–14]. Over the last decade, a number of independently conducted studies have demonstrated the antimicrobial activity of *α*-MSH as well as its C-terminals (containing Lys-Pro-Val) against a wide range of microbes including* Candida albicans*,* Escherichia coli*, and* Staphylococcus aureus* [15–19].

A major hurdle faced in developing drugs based on natural AMPs is the occurrence of inflammation. Due to this, the development of new anti-infective agents with both antimicrobial and anti-inflammatory properties has got serious attention in recent years. This is where *α*-MSH comes across as unique compared to other natural AMPs—it is endogenous anti-inflammatory, antipyretic neuropeptide combined with antimicrobial properties [11]. We present here the in-depth analysis of the current evidences supporting the direct antimicrobial and immunomodulatory potential of *α*-MSH and analogues. This paper is aimed at emphasizing the future perspectives of *α*-MSH as a therapeutic against microbial infection in humans.

## 2. Classical AMPs from Humans

It would not be inappropriate to describe AMPs as an ancient evolutionary arsenal deployed by all life forms. They have been instrumental throughout the evolution of multicellular organisms in defending against the ever-evolving microbes [8–10]. AMPs are widely secreted throughout the body, that is, epithelial, epidermal, immune cells, mucosal surfaces, and so forth. Besides being involved in the direct killing of microbes, they also play an indirect role in clearing them through immunomodulatory activities [20, 21].

Human AMPs are broadly grouped into two families: defensins and cathelicidins. Defensins are widely present in vertebrates including primates, rodents, marsupials, and mammals. Generally, they are 29 to 30 amino acid long and up to 5 kDa and contain 6-cysteines, forming 3 disulphide bonds [22]. They are further subgrouped into *α*-defensins and *β*-defensins, based on the arrangement of their disulphide bridges. There is also a third class of defensins, found in old-world monkeys, called *θ*-defensins. In humans, six different *α*-defensins are present. Four of these were found in the granules of neutrophils and named as human neutrophil peptides (HNP) 1–4 [22, 23].

The other two human *α*-defensins (HD)—HD5 and HD6—are expressed in the paneth cells of the intestinal tract [24]. The human *β*-defensins (HBD) are of four types: HBD1–4. HBD1 is expressed constitutively in most tissues, including the epithelial cells and salivary and mammary glands.

The second human AMP family is cathelicidins. Besides humans, cathelicidins are also found in cows, sheep, guinea pigs, rabbits, mice, and primates. The 37 amino acid long; C-terminal peptide of human cathelicidin antimicrobial protein (hCAP18) is called LL-37 because it begins with two leucines. It is a broad spectrum AMP, produced by the epithelial cells, immune cells, and body fluids such as gastric juices and saliva. The expression of LL-37 increases in response to infections and inflammatory conditions of the skin, such as atopic dermatitis and psoriasis [25]. Besides the direct killing of bacteria, LL-37 also clears pathogens by acting as opsonins, leading to the phagocytosis of LL-37 coated bacteria by macrophages [26].

## 3. Mode of Action and Functions of AMPs

AMPs are the endogenous and integral molecules of the innate immune system and represent the first line of host defense. The cationic and amphiphilic characteristics of AMPs favor their interaction with the anionic microbial membrane, which has differentiating features from the mammalian cell membrane. For example, the bacterial membranes are negative in charge due to the presence of anionic phospholipids (PG, PS, and CL), lipoteichoic acid (LTA), and teichoic acid (TA) in Gram-positive bacteria and lipopolysaccharides (LPS) in Gram-negative bacteria [34]. Another fundamental difference between the microbial and mammalian membranes is that the former have a greater potential difference across the plasma membrane bilayer (−130 mV to −150 mV) compared to the mammalian cell membranes (−90 mV to −110 mV). This further increases the electronegativity of bacterial membrane [35].

Thus AMPs kill microbes directly by perforation of their membrane through electrostatic interaction [8, 9]. The hydrophilic domain, that is, cationic amino acids of AMPs, binds electrostatically with anionic bacterial membrane and its hydrophobic part helps in penetration in to the lipid part of the bacterial membrane [10]. The interaction of the AMPs with the bacterial membrane happens in the following four steps as described by Shai Matsuzaki and Huang (SMH) model [36–38]. In the first step, the peptides acquire the secondary structure if not preadopted upon binding with membrane in an amphiphilic manner where positive and hydrophobic amino acids are separated in distinct domains and interact with negative heads of the lipids on bacterial membrane [39]. Secondly, these lipids are removed from membrane and thickness of membrane is thus reduced, allowing aggregation of more AMPs at the lipid displaced site of membrane leading to high peptide/lipid ratio. Thirdly, AMPs insert perpendicularly into the membrane through channel or pore formation. In the fourth and last step they perform final bacterial killing either by loss of membrane potential and ionic loss followed by cell death (e.g., magainin and alamethicin) [36] or by travelling across the membrane and targeting other intracellular pathways (e.g., buforin II and mersacidin) of bacterial machinery [40]. In some cases both processes occur simultaneously for the same kind of peptide (e.g., nisin) [41]. To describe peptide insertion and pore formation, three models have been proposed: (1) carpet model, (2) barrel-stave model, and (3) toroidal-pore model [36–38].

In the higher organisms, however, AMPs not only participate in the immediate innate immunity to infections (direct killing), they also establish a connection between innate and adaptive immunity and function as crucial signaling mediators in host immunity and inflammation [20, 42]. On exposure to pathogens, the innate immunity gets triggered through receptor activation. For example, toll-like receptors (TLRs), IL-1R and G-protein coupled receptors (GPCRs), and nod-like receptors (NLRs) are collectively known as pathogen-recognizing receptors (PRRs) [21]. They recognize and bind to pathogenic antigens like LPS and endotoxins [23]. The activation of these receptors eventually leads to the stimulation of several genes of immune cells and skin cells (encoded for AMPs). These cells release AMPs to directly encounter the microbes and clear the infection [24].

When the AMPs alone are not sufficient to clear all the microbes, they execute immune modulation through following effector mechanisms, (i) the downregulation of proinflammatory cytokines, (ii) immune cell differentiation and polarization, and (iii) chemoattraction of the immune cells [20, 21, 28, 42, 43]. All these effector mechanisms are interdependent. For instance, defensins stimulate the production of macrophage proinflammatory cytokine to stimulate the migration of antigen-presenting cells (APCs) to the site of infection [35]. It has also been observed that AMPs perform their antimicrobial role by interacting with TLRs in drosophila and its homologues in humans [44]. Moreover, studies have shown that AMPs (i.e., LL-37 and HBD2) have an anti-inflammatory role in combating various inflammatory diseases such as psoriasis, Crohn's disease, and morbus Kostmann disease [25, 42].

## 4. Expanding the Repertoire of Human AMPs

Besides defensins and cathelicidins, several other peptides of humans are recognized as important antimicrobial agents. For example, histatins in saliva, thrombin-induced platelet microbicidal protein (tPMP-1) in blood, dermcidin in sweat, hepcidin in liver, lactoferrin in milk, and lipocalin in urinary tract [10]. Moreover, many immunomodulatory peptides, including chemokines, have recently been identified exhibiting direct antimicrobial properties. Such antimicrobial chemokines are termed as kinocidins [20, 21]. Several neuropeptides, such as neurokinin 1 (NK1), neuropeptide y (NPY), and proenkephalin A (PEA), are reported to have antimicrobial properties [45, 46]. In addition, the anti-inflammatory and antipyretic properties of melanocortin peptides and their widespread presence are indicators of their involvement in host defense [47, 48]. A peptide called *α*-MSH from melanocortin family has been reported for its broad-spectrum antimicrobial effects [14, 49, 50].

The above families of peptides have tremendous antimicrobial potential; however, they are not yet widely recognized as members of host defense peptides [35]. Cathepsin G, human neutrophil elastase, and azurocidin stored in azurophils, collectively called serprocidins, have also been found killing bacteria directly [28].  Table 1 shows the sequence of all major host defense peptides from humans.

## 5. Melanocortin Peptides and Their Biochemistry

Melanocortin (MC) peptides are derived from proopiomelanocortin (POMC) and include adrenocorticotropic hormone (ACTH); *β*-lipoprotein hormone (*β*-LPH); *α*-, *β*-, and *γ*-MSH; and corticotrophin-like intermediate peptide (CLIP) [3, 4]. In higher vertebrates, the POMC gene is expressed predominantly in the pituitary (intermediate lobe) gland, but its expression has been detected widely in peripheral tissues as well. Melanocortin is an ancient peptide family that has existed since the Paleozoic Era, and the expression of POMC has been recognized in lamprey, an earliest vertebrate [29].

All melanocortins work through interaction with melanocortin receptors (MCRs); thus MC peptides have in common a four amino acid long core sequence—His-Phe-Arg-Trp (HFRW)—which works as both the message sequence and the address sequence and activates the MCRs [30, 31]. MCRs belong to the rhodopsin family of G protein coupled receptors (GPCRs) [32]. There are five MCRs: MC1R, MC2R, MC3R, MC4R, and MC5R. Except MC2R, the other four are present in the brain and can be activated by MSHs or ACTH. MC2R, on the other hand, expresses in adrenal cells, activated only by ACTH and implicated in corticotropic activities [30, 32]. The distribution of MCRs and their agonists, functions, and the signaling system have been illustrated in Table 2. MC1R, MC3R, MC4R, and MC5R take part in anti-inflammation, thus protecting the brain and peripheral tissues from acute inflammatory injuries [4, 11].

## 6. Alpha-Melanocyte Stimulating Hormone (***α***-MSH)

*α*-MSH is generated as a result of post-translational processing of POMC by serine proteinases called prohormone convertases 1 and 2 (PC1 and PC2). PC1 cleaves POMC at arginine or lysine residue and produces ACTH, *γ*-MSH, and *β*-LPH [3]. Thereafter, a second proteinase PC2 cleaves ACTH to give CLIP and *α*-MSH. *β*-MSH is produced from the cleavage of *β*-LPH [4]. *α*-MSH is a 13 amino acid (AC-S^1^Y^2^S^3^M^4^E^5^H^6^F^7^R^8^W^9^G^10^K^11^P^12^V^13^-COOH) long, cationic peptide [31]. The N-terminal of *α*-MSH is acetylated and C-terminal is amidated after posttranslational modification. *α*-MSH is widely secreted from pituitary gland of central nervous system (CNS) to several peripheral cells [4, 30].

Thus, *α*-MSH performs two types of biological functions based on its (i) hormonal effects such as melanin synthesis, secretion of sebum, regulation of temperature, control of pain, and regulation of behavior involving sex, feeding, and learning and (ii) immunomodulatory effects such as the cure of several inflammatory conditions of brain (meningitis, vasculitis, etc.) and peripheral organs (arthritis, colitis, etc.) and immunosuppression [2, 6, 7, 51]. In addition to this, various studies have supported the protective influence of *α*-MSH in mouse models of neurodegenerative diseases such as Alzheimer's by improving the memory [47, 52–54].

Until two decades ago this neuropeptide was primarily known as melanogenic hormone; however, later its immunomodulatory role was discovered and its anti-inflammatory effects were implicated in the cure of many inflammatory conditions [55]. The following section describes the in-depth understanding of anti-inflammatory mechanisms of *α*-MSH.

## 7. Modulation of Immune Response by ***α***-MSH to Cure Inflammatory Conditions

In mammals, as described above, besides its hormonal effects, *α*-MSH is also recognized for regulating the level of effector molecules implicated in inflammatory conditions [31, 55, 56]. *α*-MSH performs anti-inflammatory action by binding to different MCRs, such as MC1R, MCR3, and MCR5 [32]. However, it has specific affinity for MC1R, which primarily signals through the cyclic AMP-protein kinase A (cAMP-PKA) pathway to exert immunomodulatory actions [29, 51]. Inflammation is characterized by increased level of various effector molecules including proinflammatory cytokines (IL-6, IL-1, TNF-*α*, and IFN-*γ*), chemokines, and reactive oxygen and nitrogen species [57]. The expression level of *α*-MSH increases in response to the above effector molecules and thus it modulates immune reactions to reinstate immune homeostasis in both brain and peripheral organs and protect them from inflammatory damages [58–60].

The universal underlying mechanism behind the immunomodulation of *α*-MSH is the cAMP-PKA signaling pathway, followed by the inhibition of nuclear factor *κβ* (NF-*κβ*) activation, subsequently shutting down all the downstream effector proinflammatory mechanisms [61]. Stimulation of MC1R by *α*-MSH is induced in the presence of inflammatory signals, that is, proinflammatory cytokines or antigens. Activated MC1R elevates the cAMP level, which causes activation of protein kinase A (PKA) and inhibits i*κβ* kinase (IKK), thus stabilizing the i*κβ* and preventing the translocation of nuclear factor-*κβ* (NF-*κβ*) from cytosol to nucleus [11, 62]. This leads to the following downstream effector mechanisms: (i) downregulation of proinflammatory cytokines and iNOs; (ii) upregulation of anti-inflammatory/immunosuppressive cytokine (IL-10); and (iii) suppression of chemotaxis. All these reactions eventually result in the inhibition of inflammatory symptoms [63]. However, evidence has shown *α*-MSH-mediated inhibition of inflammation by involving additional signaling pathways, namely, calcium signaling pathway from MC1R and MC3R and Jak/STAT activation from MC5R [33, 56].

The inflammatory diseases cured by *α*-MSH can be classified into two categories. The first one comprises inflammatory disorders of the brain including traumatic head injury, cerebral vasospasm like subarachnoid hemorrhage (SAH), multiple sclerosis, meningitis, and brain reperfusion injury [29, 63]. The second category comprises inflammatory disorders of the peripheral organs, including the inflammatory bowel disease (Crohn's disease and ulcerative colitis), arthritis (rheumatoid and gout), systemic inflammation (septic shock syndrome), allergic inflammation of skin, eyes, and lungs, and reperfusion injuries of the gut and heart [29, 64]. Besides, inflammation is also one of the pathologies of several neurodegenerative diseases such as Alzheimer's and others [63, 65]. The pathogenesis of Alzheimer's disease (AD) involves upregulation of several inflammatory cytokines like TNF-*α* and loss of cholinergic neurons [49, 63]. As mentioned earlier, the production of these cytokines is under the control of NF-*κβ* activation, and being a potent inhibitor of NF-*κβ* activation, *α*-MSH limits the progression of AD. Furthermore, very recently, it has been reported that *α*-MSH administration in mouse models of Alzheimer's disease prevents the GABAergic neuronal loss and thus ameliorates the cognition [47, 52–54].

Besides anti-inflammation, *α*-MSH/MC1R signaling plays a significant role in immunosuppression in case of allergic reactions [4]. Recently, a study uncovered the mechanism of immune-suppressive behavior of *α*-MSH in the case of skin inflammatory diseases. It discovered that *α*-MSH inhibits inflammation and suppresses the immune system in mouse model of psoriasis-like skin inflammation by suppressing the activation and proliferation of the effector T cells through MC1R signaling [60].

Reports have confirmed that the minimum sequence required for anti-inflammatory activity of *α*-MSH is the C-terminal tripeptide, Lys-Pro-Val (KPV) [62]. Furthermore, a dimer of C-terminal tripeptide of *α*-MSH (Cys-Lys-Pro-Val)_2_—that is, (CKPV)_2_—has been found to be a better anti-inflammatory agent than the full-length *α*-MSH and KPV for both* in vitro* and* in vivo* models of inflammation [66]. A study conducted by Capsoni et al. [7] has revealed that *α*-MSH full-length peptide and its C-terminal synthetic derivative (CKPV)_2_ can reverse the inflammatory effect of urate crystal formation in gout (a type of arthritis). Moreover, both *α*-MSH and (CKPV)_2_ possess the capacity to prevent crystal-induced chemoattraction of neutrophils [7, 66, 67].

## 8. Resemblance of ***α***-MSH to AMPs

Similar to AMPs, the anti-inflammatory activity and occurrence of *α*-MSH and its receptors in defense cells such as keratinocytes, lymphocytes, and phagocytes point towards its involvement in the clearance of infection [4]. Besides, several features of *α*-MSH overlap with AMPs [12]. Some of these are as follows. (i) *α*-MSH is a linear short peptide containing thirteen amino acids. (ii) It is positively charged and adopts the alpha helical secondary structure in bioactive form. (iii) It is a very ancient peptide that has existed since the Paleozoic Era when innate immunity was the only defense available to organisms [29]. (iv) It has a conserved nature from lower to higher vertebrates. (v) It expresses in defense organs such as skin. (vi) It is an endogenous peptide, which mitigates inflammation by immunomodulation [19, 33, 55]. (vii) The current evidences suggest that, like AMPs, *α*-MSH also exerts antimicrobial activity through membrane permeabilization [16]. (viii) It shows broad-spectrum antimicrobial activity against several* Candida* species and different bacterial genera including both Gram-positive and Gram-negative groups [14–16]. (ix) Like other AMPs, it works in synergism with the host immune system in order to fight against microbes as suggested by experiments that *α*-MSH enhances the killing activity of neutrophils [14]. (x) It acts synergistically with other conventional antibiotics and enhances their antimicrobial efficacy (this characteristic of *α*-MSH could be very useful against antibiotic resistant bacteria). (xi) Unlike defensins, which are a major class of human AMPs, *α*-MSH is salt tolerant and its antibacterial activity is intact even in the presence of physiological concentration of NaCl, MgCl_2_, and CaCl_2_ [18]. (xii) Most importantly, this neuropeptide has not shown any cytotoxicity or hemolytic activity in* in vitro* assay (the cytotoxicity has been a big obstacle in the way of clinical development of many AMPs) [68]. All the above characteristics of *α*-MSH approve its strong antimicrobial nature and it would not be inappropriate to include it in the category of AMPs of humans.

## 9. Direct Antimicrobial Activity of ***α***-MSH

*α*-MSH is a natural guard against prolonged acute-phase reactions such as inflammation and could be a unique, potential antimicrobial agent [7, 12]. Considering the increasing tolerability of bacterial and fungal pathogens towards the existing antimicrobials and the paucity of new tools to fight against them, researchers are now exploring the antimicrobial role of various immunomodulatory peptides (also known as host defense peptides (HDPs)) [14, 31]. It is well evident from previous research that *α*-MSH has the potential to cure various inflammatory diseases and neurodegenerative disorders [54, 56, 60]. Moreover, its role in tuning the host immune reactions has extensively been explored in various ailments including infections [1, 64].

A decade ago, Cutuli and group reported the antimicrobial activity of *α*-MSH against* C. albicans*,* S. aureus*, and* E. coli*. They found that the peptide inhibited the formation of the germ tube and increased the level of cAMP in* C. albicans*, similar to its role in vertebrates. This group also reported that its carboxy-terminal tripeptide (KPV) and 6–13-amino-acid region (HFRWGKPV) had antimicrobial potential [14]. This shows that the essential anti-inflammatory sequence (KPV) is also essential for its direct antimicrobial efficacy. Later in 2003, Grieco et al. reported that its synthetic analogue NDP-*α*-MSH(6–13) had better candidacidal effect than native *α*-MSH [50]. Masman et al. [69] showed that the core message sequence (HFRW) of *α*-MSH possesses strong antifungal activities against* Cryptococcus neoformans*, and the antifungal activity appears to be closely related to the full-length peptide [69]. Similarly, the anticandidal activity of (CKPV)_2_ has been demonstrated in* Candida vaginitis*, both* in vitro* and* in vivo* [17, 19]. Its role in the cure of HIV patients has also been reported [4]. Further, Charnley et al. in 2008 confirmed similar antibacterial activity of C-terminal tripeptide of *α*-MSH (i.e., KPV and KP-d-V) against* E. coli* [15]. Recent studies have highlighted the antimicrobial activity of *α*-MSH against both planktonic and biofilm phenotype of* S. aureus* strains irrespective of their susceptibility to methicillin [16, 70].

Subsequently, the report emerged claiming that the C-terminal peptide region (KPV) of *α*-MSH is requisite for its antistaphylococcal effect, and the fragments of *α*-MSH devoid of KPV could not show any substantial antimicrobial activity [18]. It is interesting to note that the antimicrobial activity of *α*-MSH and its C-terminal fragments—*α*-MSH(6–13) (HFRWGKPV) and *α*-MSH(11–13) (KPV)—remains intact even in the presence of salts at their physiological concentrations, namely, NaCl (150 mM), CaCl_2_ (2 mM), and MgCl_2_ (1 mM) [18]. More recently, it has been suggested that *α*-MSH acts synergistically with antibiotics belonging to different classes. The* in vitro* pairing of *α*-MSH with gentamicin (GM), ciprofloxacin (CF), and tetracycline (TC) in a study killed the MRSA clinical strain, which was found to be strongly resistant to these antibiotics [68].

Although the antimicrobial activity of *α*-MSH is intact in the presence of blood biometrics (plasma, serum, and whole blood) and physiological salts, the peptide is not active in the presence of culture medium that is routinely used in laboratory practices [14]. To solve this obstacle, two *α*-MSH based antimicrobial analogues were derived by replacing Gly-10 in ([Dnal(2′)-7-,Phe12]-MSH-(6–13) with unnatural amino acids 2-aminoindane-2-carboxylic acid (Aic) and L-cyclohexylalanine (Cha), respectively. These had substantial killing against Gram-positive and Gram-negative bacteria and the* Candida* species in the presence of culture media [71].

The antibacterial efficacy of *α*-MSH was evaluated in mice using the intravenous staph infection model and the skin infection model. Interestingly, the* in vivo* results revealed that animals treated with *α*-MSH showed >3 log reduction in kidney bacterial counts and ≥2 log reduction in the heart, liver, spleen, and lungs. Additionally, rapid healing was observed in wounds of mice infected with* S. aureus* that were treated with *α*-MSH (M. Singh, PhD thesis, Jawaharlal Nehru University, New Delhi, India, 2013). In this connection, it is very important to mention that *α*-MSH has negligible* in vitro* hemolytic and cytotoxic effects at concentrations well above the dose required for its antibacterial effect [68].

## 10. Mechanism of Antimicrobial Activity of ***α***-MSH

As this peptide shares many properties with cationic AMPs, it is more likely to target the bacterial membrane through electrostatic interaction, leading to membrane damage and, eventually, cell death [8, 12, 27, 72]. However, the biological functions of *α*-MSH such as melanogenesis and immunomodulation in host cells are executed through the MC1R-cAMP signaling pathway [30]. Therefore, the involvement of MC1R in the antimicrobial mechanism might be another possibility. The first report by Cutuli et al. [14] clearly demonstrated that the candidacidal effect of *α*-MSH was mediated through the induction of cyclic adenosine monophosphate (cAMP), as they could not observe any linear relation between the timing of* Candida* killing and membrane leakage. They showed that a two-hour treatment of* C. albicans* with *α*-MSH greatly reduced the colony forming unit (CFU) but not the propidium iodide incorporation in the cells. However, they did observe the leakage at later time points. Cutuli et al. also observed that *α*-MSH increased the production of cAMP in* C. albicans* while the adenylyl cyclase inhibitor ddAdo partly reversed the candidacidal effect of *α*-MSH [14]. Their study also showed that the cAMP inducer, forskolin, alone caused the reduction of* C. albicans* CFU which was similar to that exerted by *α*-MSH. Therefore, they explained that the* Candida* cell death due to *α*-MSH exposure was caused by cAMP induction, a receptor mediated mechanism, and it appeared that membrane disruption was perhaps the consequence rather than the cause of* C. albicans* death. This MC1R-cAMP signaling-mediated *α*-MSH killing of* C. albicans* was further supported by another study where an analogue of *α*-MSH, [D-Nal-7,Phe-12]-MSH(6–13), exhibited an enormous increase in antimicrobial potency against* C. albicans*.

As DNal-7 is known to increase the affinity of *α*-MSH for MC1R, increased activity of melanocortins may be linked to the receptor-mediated mechanism [50]. Further, similar to the parent peptide, the dimer of C-terminal tripeptide of *α*-MSH (CKPV)_2_ also killed* C. albicans* in a rat* Candida vaginitis* model by activating the MC1R, subsequent induction of cAMP, and M2 polarization of macrophages (anti-inflammatory action) [19]. However, other studies using flow cytometry and spectrofluorimetry demonstrated that *α*-MSH and its C-terminal tripeptide containing fragments and *α*-MSH(6–13) and *α*-MSH(11–13) exert their staphylocidal activity through bacterial membrane depolarization followed by membrane permeabilization [18]. Electron microscopic images of* S. aureus* exposed with *α*-MSH and its C-terminal peptide demonstrated remarkable morphological changes including altered cell surface, incipient hole marks, rupture lines, and leakage of cell materials [18].

It appears from all the above evidence that the bacterial membrane is the major target for staphylococcal activity of *α*-MSH and related peptides. However, other targets could not be ruled out either, particularly due to the fact that the killing by *α*-MSH based peptides was very rapid, while substantial membrane disruption happened at later time points [18]. These observations suggest that either (i) the membrane damage is a secondary effect of *α*-MSH based peptide exposure and they target bacterial components other than the membranes, such as nucleic acids; or (ii) it is an important enzymatic process that leads to cell death and eventually causes membrane damage, or the occurrence of all these processes simultaneously [18]. It has been reported that a given host-defense antimicrobial peptide may use more than one mechanism for its microbicidal activity [21]. Subsequent work by Singh et al. demonstrated that *α*-MSH induced the inhibition of DNA replication and protein synthesis of* S. aureus* directly or indirectly with little effect on RNA synthesis [68].

As already described, the pronounced synergistic relation of *α*-MSH in the case of CF, GM, and TC may be due to a mechanistic analogy between these antibiotics and *α*-MSH. (CF inhibits DNA replication and GM and TC primarily decline protein synthesis.) The common killing mechanisms—either inhibition of protein synthesis or inhibition of DNA synthesis—along with other known or unknown mechanisms, such as the membrane-damaging ability of the peptide, make the combination of GM or TC or CF with *α*-MSH synergistic [68]. A scant research is done exploring the relation between cationicity of *α*-MSH and its antimicrobial efficacy. In this connection, Grieco et al. [50] have reported that the replacement of positively charged amino acid Lys at position 11 of *α*-MSH with neutral Ala diminished the anticandidal activity of *α*-MSH(6–13) by 50% [50]. However, Charnley et al. [15] showed that the cationic charge on the lysine residue is not required for the bactericidal activity of *α*-MSH(11–13). The effect of whole *α*-MSH cationicity on its antimicrobial efficacy is yet to be understood clearly.

## 11. Conclusions

In this postantibiotic era, AMPs have already created huge hopes for their host defense mechanism and several of these peptides find ways into medical practice although the numbers are less than expected. The development of AMPs as therapeutic alternatives to combat the resistant pathogenic microbes is facing problems, majorly due to the enhanced inflammatory reactions associated with them and their potential for toxicity. *α*-MSH overcomes both of these issues being anti-inflammatory and nontoxic. This endogenous neuropeptide was initially characterized as a pigment producing peptide and later received serious attention due to its potent protective and anti-inflammatory activity. *α*-MSH performs these actions by binding to centrally expressed melanocortin receptors, which subsequently coordinate numerous anti-inflammatory pathways leading to shutting down all the downstream effector proinflammatory mechanisms. Besides anti-inflammation, it also exhibited immunosuppression in case of skin-inflammatory diseases like psoriasis. The striking resemblance of this anti-inflammatory neuropeptide with cationic AMPs compelled the scientists to further explore its antimicrobial efficacy. Indeed, *α*-MSH appeared to possess potent antimicrobial activity against pathogens from different classes like* C. albicans*,* S. aureus*,* E. coli*, and more. It also adopts variable approaches to kill different microbes. For instance, it kills fungal cells through the induction of cAMP and bacterial cells by damaging the membrane [14, 18]. The C-terminal region (KPV) of *α*-MSH demands special attention for several reasons. It exhibits* in vitro* and* in vivo* anti-inflammatory activity similar to that of parent peptide without melanotropic effect. This observation removes the main obstacle in developing *α*-MSH based peptides as therapeutics, which is nothing, but its pigmentary effects and KPV are devoid of that. Moreover, this essential anti-inflammatory sequence, that is, C-terminal tripeptide (KPV) of *α*-MSH, is also essential for its direct antimicrobial efficacy. Therefore, this short molecule KPV appears to have tremendous potential to be developed as therapeutic agent as it is more suitable for clinical use and demands further research. The dimer of this short peptide, that is, (CKPV)_2_ being both anti-inflammatory and antimicrobial, is already in clinical trial and will definitely make its way to enter into medical practice in near future.

The pleiotropic effects of *α*-MSH and its C-terminal peptides, including their anti-inflammatory, immunosuppressive, antipyretic, and antimicrobial activities, are unique. These endogenous properties make them the most promising antimicrobial host defense peptides. More work is however needed to bring these peptides from the lab to clinic. First, a deeper corelation is required to be established between its anti-inflammatory and anti-infective reactions through the* in vivo* models of infections. Second, further biophysical studies are required to design a potent *α*-MSH based AMP with enhanced killing and immunomodulatory activities. Third, the possibility of resistance developing against this peptide needs to be ruled out. Fourth, overall safety profile of these peptides particularly with *α*-MSH fragments requires to be vigorously examined.

In conclusion, *α*-MSH, its analogues, and related C-terminal tripeptide with broad-spectrum antimicrobial activity combined with immunomodulating effects and no cytotoxicity could emerge as excellent therapeutic agents against resistant pathogens.

## Figures and Tables

**Table 1:** Major host defense peptides from humans and their sequences.

AMPs/structure	Site of expression	Sequence	References
*α*-Defensin, HNP1/*β*-sheet (3 s-s bonds)	Leukocytes, neutrophils	ACYCRIPACIAGERR YGTCIYQGRLWAFC C	[21, 22]
*α*-Defensin, HNP2/*β*-sheet (3 s-s bonds)	Leukocytes Neutrophils	CYCRIPACIAGERRY GTCIYQGRLWAFCC	[22, 27]
Human *α*-defensin, HNP3/*β*-sheet (3 s-s bonds)	Leukocytes Neutrophils	DCYCRIPACIAGERR YGTCIYQGRLWAFC C	[8, 22, 27]
Human *α*-defensin HNP4/*β*-sheet (3 s-s bonds)	Leukocytes Neutrophils	VCSCRLVFCRRTELR VGNCLIGGVSFTYCC TRV	[22, 28]
Human *α*-defensin, HD5/*β*-sheet (3 s-s bonds)	Intestinal tract, vaginal tract (paneth cells)	ATCYCRHGRCATRE SLSGVCEISGRLYRL CCR	[22, 24]
Human *α*-defensin, HD6/*β*-sheet (3 s-s bonds)	Intestinal tract (paneth cells)	AFTCHCRRSCYSTEY SYGTCTVMGINHRFC CL	[22, 24]
Human *β*-defensin, HBD1/*β*-sheet (3 s-s bonds)	Keratinocytes epithelium and mucous lining, salivary and mammary gland	DHYNCVSSGGQCLY SACPIFTKIQGTCYRG KACCK	[24, 28]
Human *β*-defensin, HBD2/*β*-sheet (3 s-s bonds)	Epithelium, GIT, keratinocytes	TCLKSGAICHPVFCP RRYKQIGTCGLPGTK CCKKP	[27, 28]
Human *β*-defensin HBD3/*β*-sheet (3 s-s bonds)	GIT, keratinocytes respiratory tract	GIINTLQKYYCRVRG GRCAVLSCLPKEEQI GKCSTRGRKCCRRK K	[27, 28]
Human *β*-defensin HBD4/*β*-sheet (3 s-s bonds)	Epithelium, GIT, keratinocytes	MQRLVLLLAVSLLL YQDLPVRSEFELDRI CGYGTARCRKKCRS QEYRIGRCPNTYACC LRKWDESLLNRTKP	[27, 28]
Cathelicidin LL-37/hCAP-18 Linear, *α*-helical	Epithelium, neutrophils, keratinocytes, monocytes	LLGDFFRKSKEKIGK EFKRIVQRIKDFLRNL VPRTES	[8, 21, 25]
Histatin-5/histidine rich linear, non-*α*-helical	Salivary gland	DSHAKRHHGYKRKF HEKHHSHRGY	[8]
Lactoferrin linear	Lactoferrin protein	GRRRRSVQWCAVSQ PEATKCFQWQRNMR RVRGPPVSCIKRDSPI QCIQA	[28]
Hepcidin *Β*-sheet, 4 s-s bonds	Liver	DTHFPICIFCCGCCHR SKCGMCCKT	[8]

**Table 2:** Melanocortin receptors (MCRs), their agonists, function, signaling system, and site of expression.

MCRs type	Distribution	Function	Agonists	Signaling system	References
MC1R		Pigmentation, regulation of skin physiology, anti-inflammation, pain	*α*-MSH, *β*-MSH, *γ*-MSH and its analogues (MT I, MT II)	cAMP-PKA pathway, Ca++ signaling	[1, 3, 4, 29–32]
MC2R	Adrenal cortex, murine adipocytes, skin, melanoma cells	Adrenal steroids secretion	ACTH	cAMP-PKA pathway	[1, 3, 4]
MC3R	CNS, stomach, kidneys, heart, gut, thymus, placenta	Feeding, energy homeostasis and anti-inflammation	ACTH and *γ*-MSH	cAMP-PKA, MAPK and IP/Ca++ signaling	[1, 3, 29, 33]
MC4R	CNS	Anti-inflammatory, control of feeding and sexual behaviors, energy homeostasis	*α*-MSH, ACTH	cAMP-PKA and MAPK	[1, 3, 31, 33]
MC5R	CNS and many peripheral tissues, exocrine glands, spleen, skin, lung, sexual organs, adipose tissues.	Exocrine secretion, lipolysis, regulation of body temperature	ACTH(1–24), *α*-MSH, MT I & MT II	cAMP-PKA and Jak/STAT phosphorylation pathway	[1, 3, 29, 33]

Mitogen activated protein kinase: (MAPK), Protein kinase A: (PKA), ionositol phosphate: (IP), and cyclic AMP: (cAMP).
